# 2-(2,3-Dimethyl­anilino)benzohydrazide

**DOI:** 10.1107/S1600536812032576

**Published:** 2012-07-25

**Authors:** Hoong-Kun Fun, Tze Shyang Chia, Tilal Elsaman, Mohamed I. Attia, Hatem A. Abdel-Aziz

**Affiliations:** a X-ray Crystallography Unit, School of Physics, Universiti Sains Malaysia, 11800 USM, Penang, Malaysia; b Department of Pharmaceutical Chemistry, College of Pharmacy, King Saud University, PO Box 2457, Riyadh 11451, Saudi Arabia

## Abstract

In the title compound, C_15_H_17_N_3_O, the dihedral angle between the benzene rings is 58.05 (9)°. The non-H atoms of the hydrazide group lie in a common plane (r.m.s. deviation = 0.0006 Å) and are close to coplanar with their attached benzene ring [dihedral angle = 8.02 (9)°]. An intra­molecular N—H⋯O hydrogen bond generates an *S*(6) ring motif in the mol­ecule, and a short intra­molecular contact (H⋯H = 1.88 Å) is also observed. In the crystal, mol­ecules are linked by pairs of N—H⋯N hydrogen bonds into inversion dimers. The crystal packing also features C—H⋯π inter­actions.

## Related literature

For the biological activity of fenamates, see: Boschelli *et al.* (1990 ▶); Reddy *et al.* (2010 ▶); Aboul-Fadl *et al.* (2011 ▶). For the synthesis, see: Reddy *et al.* (2010 ▶); Aboul-Fadl *et al.* (2011 ▶). For a related structure, see: Bhat *et al.* (2012 ▶). For hydrogen-bond motifs, see: Bernstein *et al.* (1995 ▶). For reference bond-length data, see: Allen *et al.* (1987 ▶). For the stability of the temperature controller used for the data collection, see: Cosier & Glazer (1986 ▶).
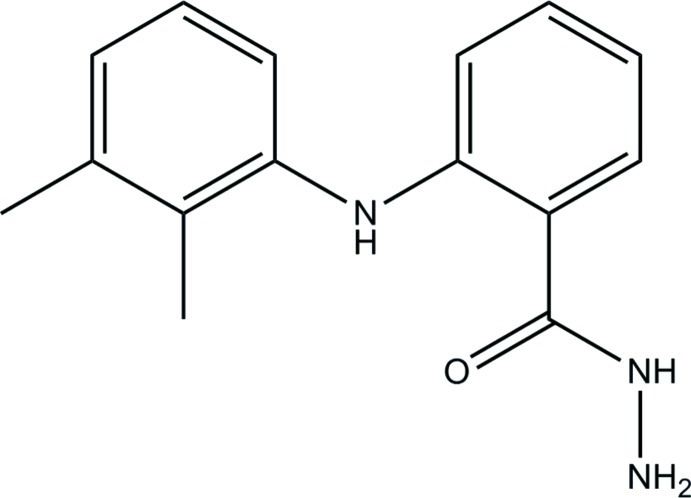


## Experimental

### 

#### Crystal data

C_15_H_17_N_3_O
*M* *_r_* = 255.32
Triclinic, 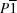

*a* = 6.9092 (8) Å
*b* = 6.9609 (7) Å
*c* = 14.9458 (15) Å
α = 81.562 (2)°
β = 81.328 (2)°
γ = 66.269 (2)°
*V* = 647.56 (12) Å^3^
*Z* = 2
Mo *K*α radiation
μ = 0.09 mm^−1^
*T* = 100 K
0.28 × 0.18 × 0.13 mm

#### Data collection

Bruker APEX DUO CCD diffractometer
Absorption correction: multi-scan (*SADABS*; Bruker, 2009 ▶) *T*_min_ = 0.977, *T*_max_ = 0.989
8491 measured reflections
2218 independent reflections
1826 reflections with *I* > 2σ(*I*)
*R*_int_ = 0.028

#### Refinement

*R*[*F* ^2^ > 2σ(*F* ^2^)] = 0.039
*wR*(*F* ^2^) = 0.108
*S* = 1.05
2218 reflections
190 parameters
H atoms treated by a mixture of independent and constrained refinement
Δρ_max_ = 0.21 e Å^−3^
Δρ_min_ = −0.20 e Å^−3^

### 

Data collection: *APEX2* (Bruker, 2009 ▶); cell refinement: *SAINT* (Bruker, 2009 ▶); data reduction: *SAINT*; program(s) used to solve structure: *SHELXTL* (Sheldrick, 2008 ▶); program(s) used to refine structure: *SHELXTL*; molecular graphics: *SHELXTL*; software used to prepare material for publication: *SHELXTL* and *PLATON* (Spek, 2009 ▶).

## Supplementary Material

Crystal structure: contains datablock(s) global, I. DOI: 10.1107/S1600536812032576/hb6900sup1.cif

Structure factors: contains datablock(s) I. DOI: 10.1107/S1600536812032576/hb6900Isup2.hkl

Supplementary material file. DOI: 10.1107/S1600536812032576/hb6900Isup3.cml

Additional supplementary materials:  crystallographic information; 3D view; checkCIF report

## Figures and Tables

**Table 1:** Hydrogen-bond geometry (Å, °) *Cg*1 is the centroid of the C1–C6 ring.

*D*—H⋯*A*	*D*—H	H⋯*A*	*D*⋯*A*	*D*—H⋯*A*
N2—H1N2⋯N3^i^	0.89 (2)	2.29 (2)	3.129 (2)	158.4 (19)
N1—H1N1⋯O1	0.89 (2)	1.90 (2)	2.6667 (19)	143.4 (18)
C11—H11*A*⋯*Cg*1^ii^	0.93	2.58	3.303 (2)	135
C14—H14*C*⋯*Cg*1^iii^	0.96	2.77	3.535 (2)	137

Symmetry codes: (i) 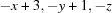; (ii) 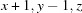; (iii) 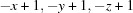.

**Fig. 1.**
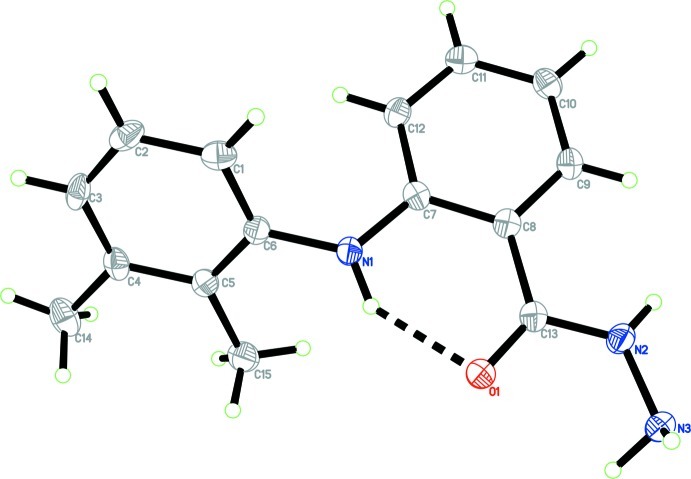
The molecular structure of the title compound with 50% probability displacement ellipsoids. The dashed line represents the intramolecular N—H···O hydrogen bond.

**Fig. 2.**
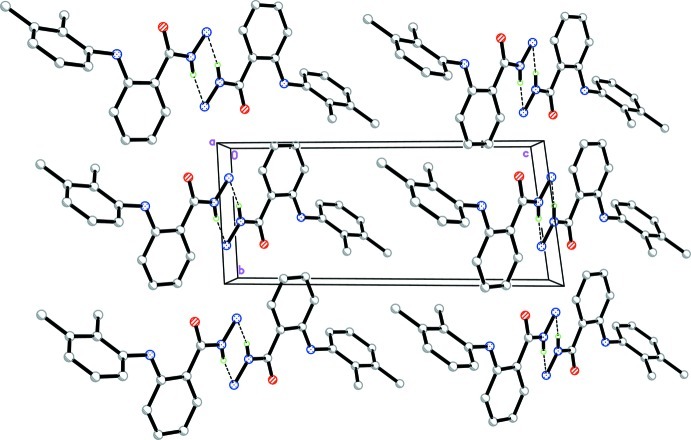
The crystal packing of the title compound viewed along the a axis. The dashed lines represent the hydrogen bonds. For clarity sake, hydrogen atoms not involved in hydrogen bonding have been omitted.

## supplementary crystallographic information

### Comment

Mefenamic acid (MFA), *N*-(2,3-xylyl)anthranilic acid and meclofenamic acid (MCFA) are derivatives of fenamates. They are non-steroidal anti-inflammatory drugs (NSAIDs) used as potent analgesic and anti-inflammatory agents in the treatment of osteoarthritis and rheumatoid arthritis (Boschelli *et al.*, 1990; Reddy *et al.*, 2010; Aboul-Fadl *et al.*, 2011). In view of the importance of the hydrazide of fenamic acid as an active synthon in the synthesis of compounds with biological interests (Reddy *et al.*, 2010; Bhat *et al.*, 2012), we report herein the crystal structure of the title compound.

The molecular structure of the title compound is shown in Fig. 1. The C1–C6 benzene ring makes a dihedral angle of 58.05 (9)° with the C7–C12 benzene ring. The non-H atoms of hydrazide group (O1/N2/N3/C13) lie nearly on a plane [r.m.s. deviation = 0.0006 Å] and are nearly coplanar with the attached C7–C12 benzene ring as indicated by the dihedral angle of 8.02 (9)°. An intramolecular N1—H1N1···O1 hydrogen bond generates an S(6) ring motif (Bernstein *et al.*, 1995) in the molecule. Bond lengths (Allen *et al.*, 1987) and angles are within normal ranges and are comparable to those in a related structure (Bhat *et al.*, 2012).

In the crystal (Fig. 2), the molecules are linked by pairs of intermolecular N2—H1N2···N3 hydrogen bond into inversion dimers. The crystal packing is further stabilized by C—H···π interactions (Table 1), involving *Cg*1 which is the centroid of C1–C6 ring. A short intramolecular contact [H1N2···H9A = 1.88 Å] is also observed.

### Experimental

The title compound was prepared by the reaction of the methyl ester of fenamic acid with hydrazine hydrate or with the direct reaction of fenamic acid with hydrazine hydrate under microwave irritation (Reddy *et al.*, 2010; Aboul-Fadl *et al.*, 2011). Brown blocks were grown from the slow evaporation of a methanol solution.

### Refinement

The N-bound H atoms were located in a difference Fourier map and refined freely [N—H = 0.89 (2), 0.93 (3) and 0.963 (19) Å]. The remaining H atoms were positioned geometrically [C—H = 0.93 and 0.96 Å] and refined with *U*_iso_(H) = 1.2 or 1.5*U*_eq_(C). A rotating group model was applied to the methyl groups. Five outliers, (213), (417), (533), (300) and (532), were omitted in the final refinement.

### Crystal data

**Table:**

C_15_H_17_N_3_O	*Z* = 2
*M**_r_* = 255.32	*F*(000) = 272
Triclinic, *P*1	*D*_x_ = 1.309 Mg m^−^^3^
Hall symbol: -P 1	Mo *K*α radiation, λ = 0.71073 Å
*a* = 6.9092 (8) Å	Cell parameters from 4059 reflections
*b* = 6.9609 (7) Å	θ = 2.8–30.3°
*c* = 14.9458 (15) Å	µ = 0.09 mm^−^^1^
α = 81.562 (2)°	*T* = 100 K
β = 81.328 (2)°	Block, brown
γ = 66.269 (2)°	0.28 × 0.18 × 0.13 mm
*V* = 647.56 (12) Å^3^	

### Data collection

**Table:**

Bruker APEX DUO CCD diffractometer	2218 independent reflections
Radiation source: fine-focus sealed tube	1826 reflections with *I* > 2σ(*I*)
Graphite monochromator	*R*_int_ = 0.028
φ and ω scans	θ_max_ = 25.0°, θ_min_ = 1.4°
Absorption correction: multi-scan (*SADABS*; Bruker, 2009)	*h* = −8→8
*T*_min_ = 0.977, *T*_max_ = 0.989	*k* = −8→8
8491 measured reflections	*l* = −17→17

### Refinement

**Table:**

Refinement on *F*^2^	Primary atom site location: structure-invariant direct methods
Least-squares matrix: full	Secondary atom site location: difference Fourier map
*R*[*F*^2^ > 2σ(*F*^2^)] = 0.039	Hydrogen site location: inferred from neighbouring sites
*wR*(*F*^2^) = 0.108	H atoms treated by a mixture of independent and constrained refinement
*S* = 1.05	*w* = 1/[σ^2^(*F*_o_^2^) + (0.042*P*)^2^ + 0.4394*P*] where *P* = (*F*_o_^2^ + 2*F*_c_^2^)/3
2218 reflections	(Δ/σ)_max_ < 0.001
190 parameters	Δρ_max_ = 0.21 e Å^−^^3^
0 restraints	Δρ_min_ = −0.20 e Å^−^^3^

### Special details

**Table:**

Experimental. The crystal was placed in the cold stream of an Oxford Cryosystems Cobra open-flow nitrogen cryostat (Cosier & Glazer, 1986) operating at 100.0 (1) K.
Geometry. All e.s.d.'s (except the e.s.d. in the dihedral angle between two l.s. planes) are estimated using the full covariance matrix. The cell e.s.d.'s are taken into account individually in the estimation of e.s.d.'s in distances, angles and torsion angles; correlations between e.s.d.'s in cell parameters are only used when they are defined by crystal symmetry. An approximate (isotropic) treatment of cell e.s.d.'s is used for estimating e.s.d.'s involving l.s. planes.
Refinement. Refinement of *F*^2^ against ALL reflections. The weighted *R*-factor *wR* and goodness of fit *S* are based on *F*^2^, conventional *R*-factors *R* are based on *F*, with *F* set to zero for negative *F*^2^. The threshold expression of *F*^2^ > σ(*F*^2^) is used only for calculating *R*-factors(gt) *etc*. and is not relevant to the choice of reflections for refinement. *R*-factors based on *F*^2^ are statistically about twice as large as those based on *F*, and *R*-factors based on ALL data will be even larger.

### Fractional atomic coordinates and isotropic or equivalent isotropic displacement parameters (Å^2^)

**Table:**

	*x*	*y*	*z*	*U*_iso_*/*U*_eq_	
O1	0.91711 (19)	0.74672 (18)	0.09083 (8)	0.0228 (3)	
N1	0.7359 (2)	0.5409 (2)	0.21691 (10)	0.0232 (4)	
N2	1.2514 (3)	0.5701 (2)	0.03071 (10)	0.0228 (4)	
N3	1.2742 (2)	0.7493 (2)	−0.01967 (10)	0.0220 (3)	
C1	0.4439 (3)	0.4330 (3)	0.27761 (11)	0.0214 (4)	
H1A	0.4763	0.3502	0.2296	0.026*	
C2	0.2757 (3)	0.4418 (3)	0.34225 (12)	0.0235 (4)	
H2A	0.1957	0.3636	0.3385	0.028*	
C3	0.2270 (3)	0.5677 (3)	0.41256 (11)	0.0228 (4)	
H3A	0.1144	0.5724	0.4562	0.027*	
C4	0.3424 (3)	0.6870 (3)	0.41951 (11)	0.0202 (4)	
C5	0.5160 (3)	0.6764 (3)	0.35493 (11)	0.0181 (4)	
C6	0.5649 (3)	0.5483 (2)	0.28435 (11)	0.0181 (4)	
C7	0.9115 (3)	0.3622 (3)	0.19813 (10)	0.0173 (4)	
C8	1.0787 (3)	0.3723 (3)	0.13174 (10)	0.0173 (4)	
C9	1.2500 (3)	0.1835 (3)	0.11310 (11)	0.0205 (4)	
H9A	1.3578	0.1879	0.0684	0.025*	
C10	1.2652 (3)	−0.0080 (3)	0.15836 (11)	0.0219 (4)	
H10A	1.3814	−0.1305	0.1445	0.026*	
C11	1.1052 (3)	−0.0163 (3)	0.22493 (11)	0.0211 (4)	
H11A	1.1149	−0.1448	0.2565	0.025*	
C12	0.9318 (3)	0.1645 (3)	0.24464 (11)	0.0196 (4)	
H12A	0.8258	0.1562	0.2896	0.023*	
C13	1.0721 (3)	0.5779 (3)	0.08386 (10)	0.0178 (4)	
C14	0.2827 (3)	0.8260 (3)	0.49557 (12)	0.0297 (4)	
H14A	0.1620	0.8139	0.5333	0.045*	
H14B	0.2479	0.9698	0.4707	0.045*	
H14C	0.4002	0.7831	0.5313	0.045*	
C15	0.6429 (3)	0.8056 (3)	0.36060 (13)	0.0272 (4)	
H15A	0.7813	0.7434	0.3285	0.041*	
H15B	0.6571	0.8093	0.4232	0.041*	
H15C	0.5712	0.9465	0.3338	0.041*	
H1N2	1.370 (3)	0.454 (3)	0.0319 (13)	0.027 (5)*	
H1N1	0.756 (3)	0.654 (3)	0.1890 (14)	0.032 (6)*	
H2N3	1.204 (3)	0.867 (3)	0.0165 (13)	0.022 (5)*	
H1N3	1.188 (4)	0.795 (4)	−0.0667 (17)	0.053 (7)*	

### Atomic displacement parameters (Å^2^)

**Table:**

	*U*^11^	*U*^22^	*U*^33^	*U*^12^	*U*^13^	*U*^23^
O1	0.0206 (7)	0.0180 (6)	0.0261 (6)	−0.0056 (5)	0.0023 (5)	−0.0010 (5)
N1	0.0212 (9)	0.0164 (8)	0.0258 (8)	−0.0049 (6)	0.0064 (6)	0.0002 (6)
N2	0.0184 (9)	0.0202 (8)	0.0263 (8)	−0.0068 (7)	0.0021 (6)	0.0018 (6)
N3	0.0226 (9)	0.0212 (8)	0.0223 (8)	−0.0101 (7)	0.0000 (6)	0.0001 (6)
C1	0.0243 (10)	0.0181 (8)	0.0220 (8)	−0.0077 (7)	−0.0084 (7)	0.0022 (7)
C2	0.0195 (10)	0.0224 (9)	0.0308 (9)	−0.0115 (8)	−0.0090 (7)	0.0073 (7)
C3	0.0121 (9)	0.0278 (10)	0.0222 (9)	−0.0048 (8)	−0.0016 (7)	0.0079 (7)
C4	0.0141 (9)	0.0215 (9)	0.0176 (8)	0.0000 (7)	−0.0042 (6)	0.0024 (6)
C5	0.0130 (9)	0.0170 (8)	0.0217 (8)	−0.0032 (7)	−0.0054 (6)	0.0019 (6)
C6	0.0151 (9)	0.0165 (8)	0.0189 (8)	−0.0039 (7)	−0.0016 (6)	0.0033 (6)
C7	0.0163 (9)	0.0191 (8)	0.0160 (8)	−0.0054 (7)	−0.0034 (6)	−0.0030 (6)
C8	0.0171 (9)	0.0208 (9)	0.0150 (8)	−0.0077 (7)	−0.0043 (6)	−0.0016 (6)
C9	0.0174 (9)	0.0230 (9)	0.0193 (8)	−0.0063 (7)	−0.0004 (7)	−0.0026 (7)
C10	0.0188 (10)	0.0197 (9)	0.0232 (9)	−0.0024 (7)	−0.0039 (7)	−0.0026 (7)
C11	0.0240 (10)	0.0195 (9)	0.0192 (8)	−0.0074 (8)	−0.0077 (7)	0.0026 (6)
C12	0.0192 (9)	0.0225 (9)	0.0172 (8)	−0.0088 (7)	−0.0016 (6)	−0.0002 (6)
C13	0.0176 (9)	0.0218 (9)	0.0155 (8)	−0.0080 (8)	−0.0031 (6)	−0.0034 (6)
C14	0.0242 (11)	0.0312 (10)	0.0246 (9)	−0.0001 (8)	−0.0051 (8)	−0.0039 (8)
C15	0.0206 (10)	0.0234 (9)	0.0385 (11)	−0.0083 (8)	−0.0042 (8)	−0.0051 (8)

### Geometric parameters (Å, º)

**Table:**

O1—C13	1.236 (2)	C5—C15	1.505 (2)
N1—C7	1.372 (2)	C7—C12	1.411 (2)
N1—C6	1.422 (2)	C7—C8	1.422 (2)
N1—H1N1	0.89 (2)	C8—C9	1.401 (2)
N2—C13	1.353 (2)	C8—C13	1.489 (2)
N2—N3	1.412 (2)	C9—C10	1.375 (2)
N2—H1N2	0.89 (2)	C9—H9A	0.9300
N3—H2N3	0.963 (19)	C10—C11	1.385 (2)
N3—H1N3	0.93 (3)	C10—H10A	0.9300
C1—C2	1.382 (3)	C11—C12	1.377 (2)
C1—C6	1.394 (2)	C11—H11A	0.9300
C1—H1A	0.9300	C12—H12A	0.9300
C2—C3	1.383 (3)	C14—H14A	0.9600
C2—H2A	0.9300	C14—H14B	0.9600
C3—C4	1.385 (2)	C14—H14C	0.9600
C3—H3A	0.9300	C15—H15A	0.9600
C4—C5	1.406 (2)	C15—H15B	0.9600
C4—C14	1.503 (2)	C15—H15C	0.9600
C5—C6	1.395 (2)		
			
C7—N1—C6	124.88 (14)	C9—C8—C7	118.05 (15)
C7—N1—H1N1	110.0 (14)	C9—C8—C13	121.02 (15)
C6—N1—H1N1	124.1 (13)	C7—C8—C13	120.93 (15)
C13—N2—N3	123.16 (15)	C10—C9—C8	122.59 (16)
C13—N2—H1N2	121.2 (13)	C10—C9—H9A	118.7
N3—N2—H1N2	115.0 (13)	C8—C9—H9A	118.7
N2—N3—H2N3	108.0 (11)	C9—C10—C11	119.13 (16)
N2—N3—H1N3	109.7 (15)	C9—C10—H10A	120.4
H2N3—N3—H1N3	99.5 (19)	C11—C10—H10A	120.4
C2—C1—C6	119.93 (16)	C12—C11—C10	120.42 (16)
C2—C1—H1A	120.0	C12—C11—H11A	119.8
C6—C1—H1A	120.0	C10—C11—H11A	119.8
C1—C2—C3	119.54 (16)	C11—C12—C7	121.37 (15)
C1—C2—H2A	120.2	C11—C12—H12A	119.3
C3—C2—H2A	120.2	C7—C12—H12A	119.3
C2—C3—C4	121.49 (15)	O1—C13—N2	120.69 (15)
C2—C3—H3A	119.3	O1—C13—C8	124.06 (15)
C4—C3—H3A	119.3	N2—C13—C8	115.25 (15)
C3—C4—C5	119.36 (15)	C4—C14—H14A	109.5
C3—C4—C14	120.26 (16)	C4—C14—H14B	109.5
C5—C4—C14	120.38 (16)	H14A—C14—H14B	109.5
C6—C5—C4	118.89 (15)	C4—C14—H14C	109.5
C6—C5—C15	120.69 (15)	H14A—C14—H14C	109.5
C4—C5—C15	120.39 (15)	H14B—C14—H14C	109.5
C1—C6—C5	120.76 (15)	C5—C15—H15A	109.5
C1—C6—N1	119.64 (15)	C5—C15—H15B	109.5
C5—C6—N1	119.57 (15)	H15A—C15—H15B	109.5
N1—C7—C12	120.95 (15)	C5—C15—H15C	109.5
N1—C7—C8	120.68 (15)	H15A—C15—H15C	109.5
C12—C7—C8	118.37 (15)	H15B—C15—H15C	109.5
			
C6—C1—C2—C3	0.9 (2)	N1—C7—C8—C9	−177.64 (15)
C1—C2—C3—C4	0.5 (3)	C12—C7—C8—C9	2.9 (2)
C2—C3—C4—C5	−1.5 (2)	N1—C7—C8—C13	2.7 (2)
C2—C3—C4—C14	178.65 (15)	C12—C7—C8—C13	−176.72 (14)
C3—C4—C5—C6	1.2 (2)	C7—C8—C9—C10	−2.1 (2)
C14—C4—C5—C6	−178.98 (15)	C13—C8—C9—C10	177.53 (15)
C3—C4—C5—C15	179.57 (16)	C8—C9—C10—C11	0.2 (3)
C14—C4—C5—C15	−0.6 (2)	C9—C10—C11—C12	0.9 (2)
C2—C1—C6—C5	−1.2 (2)	C10—C11—C12—C7	0.0 (2)
C2—C1—C6—N1	−179.12 (15)	N1—C7—C12—C11	178.61 (16)
C4—C5—C6—C1	0.1 (2)	C8—C7—C12—C11	−2.0 (2)
C15—C5—C6—C1	−178.22 (15)	N3—N2—C13—O1	−0.2 (2)
C4—C5—C6—N1	178.07 (14)	N3—N2—C13—C8	−179.91 (14)
C15—C5—C6—N1	−0.3 (2)	C9—C8—C13—O1	172.87 (15)
C7—N1—C6—C1	−61.6 (2)	C7—C8—C13—O1	−7.5 (2)
C7—N1—C6—C5	120.45 (18)	C9—C8—C13—N2	−7.4 (2)
C6—N1—C7—C12	2.0 (3)	C7—C8—C13—N2	172.23 (14)
C6—N1—C7—C8	−177.41 (15)		

### Hydrogen-bond geometry (Å, º)

Cg1 is the centroid of the C1–C6 ring.

**Table:**

*D*—H···*A*	*D*—H	H···*A*	*D*···*A*	*D*—H···*A*
N2—H1*N*2···N3^i^	0.89 (2)	2.29 (2)	3.129 (2)	158.4 (19)
N1—H1*N*1···O1	0.89 (2)	1.90 (2)	2.6667 (19)	143.4 (18)
C11—H11*A*···*Cg*1^ii^	0.93	2.58	3.303 (2)	135
C14—H14*C*···*Cg*1^iii^	0.96	2.77	3.535 (2)	137

Symmetry codes: (i) −*x*+3, −*y*+1, −*z*; (ii) *x*+1, *y*−1, *z*; (iii) −*x*+1, −*y*+1, −*z*+1.
